# Spot Spine, a freely available ImageJ plugin for 3D detection and morphological analysis of dendritic spines

**DOI:** 10.12688/f1000research.146327.2

**Published:** 2024-09-05

**Authors:** Jean-Francois Gilles, Philippe Mailly, Tiago Ferreira, Thomas Boudier, Nicolas Heck

**Affiliations:** 1 Institut de Biologie Paris Seine, CNRS, Sorbonne Universite, Paris, Île-de-France, France; 2 CRIB, CNRS, College de France, Paris, Île-de-France, France; 3 Howard Hughes Medical Institute Janelia Farm Research Campus, Ashburn, Virginia, USA; 4 INRIA, CNRS, Ecole Centrale Méditerranée, University of Côte d'Azur, Nice, Provence-Alpes-Côte d'Azur, France; 5 Neuroscience Paris Seine, CNRS, Sorbonne Universite, Paris, Île-de-France, France

**Keywords:** Dendritic spine, neuronal morphology, neuroanatomy, synapse, image analysis, ImageJ, Fiji

## Abstract

**Background:**

Dendritic spines are tiny protrusions found along the dendrites of neurons, and their number is a measure of the density of synaptic connections. Altered density and morphology is observed in several pathologies, and spine formation as well as morphological changes correlate with learning and memory. The detection of spines in microscopy images and the analysis of their morphology is therefore a prerequisite for many studies. We have developed a new open-source, freely available, plugin for ImageJ/FIJI, called Spot Spine, that allows detection and morphological measurements of spines in three dimensional images.

**Method:**

Local maxima are detected in spine heads, and the intensity distribution around the local maximum is computed to perform the segmentation of each spine head. Spine necks are then traced from the spine head to the dendrite. Several parameters can be set to optimize detection and segmentation, and manual correction gives further control over the result of the process.

**Results:**

The plugin allows the analysis of images of dendrites obtained with various labeling and imaging methods. Quantitative measurements are retrieved including spine head volume and surface, and neck length.

**Conclusion:**

The plugin and instructions for use are available at https://imagej.net/plugins/spot-spine.

## Introduction

Dendritic spines are small protrusions distributed along the dendrites of neurons. Since each spine corresponds to a glutamatergic synapse, the density of spines located along the dendrite is an estimate of the density of neuronal connectivity. Spine density measurement is thus pivotal for assessing connectivity changes during development, upon synaptic plasticity and learning, as well as in the context of psychiatric diseases ( Holtmaat and Svoboda, 2009; Penzes *et al.*, 2011; Ma and Zuo, 2022; Heck and Santos, 2023). The morphology of dendritic spines, which are composed of a neck and a head, is another relevant parameter. The size of the head is tightly correlated with the size of the postsynapse density and synaptic current amplitude ( Noguchi *et al.*, 2011; Holler *et al.*, 2021). The length and width of the neck may have an influence on the integrative properties of the spine, since the neck represents a morphological constraint so the spine head is a functional compartment isolated from the dendritic shaft ( Adrian *et al.*, 2014; Tonnesen and Nägerl, 2016; Cornejo *et al.*, 2022). Therefore, quantification of spine density, spine head size and spine neck length are desirable for each of these parameters retrieve valuable information on neural connectivity, synapse efficacy and plasticity.

Dendritic spines however have a size that make them lay at the edge of resolution possibilities of photonic microscopy, hence detection and morphological analysis represent a challenge. Different strategies have been adopted in order to obtain reliable detection and segmentation of dendritic spines, reviewed in Mancuso *et al.* (2013) and Okabe (2020). Several algorithms have been developed, among which some have been implemented into softwares performing 3D spine detection and analysis. The first developed tools were 3DMA-neuron ( Koh *et al.*, 2002) and NeuronIQ ( Cheng *et al.*, 2007; Zhang *et al.*, 2007). The widely used NeuronStudio ( Rodriguez *et al.*, 2008) has been transferred to commercially available Neurolucida ( Dickstein *et al.*, 2016). Another commercial software is Imaris Filament Tracer module ( Swanger *et al.*, 2011; Benavides-Piccione *et al.*, 2013). Other applications for spine detection and analysis from confocal microscopy images include Spiso3D ( Mukai *et al.*, 2011), SpineLab ( Jungblut *et al.*, 2012) and a tool included in the FARSIGHT project ( Yuan *et al.*, 2009). More recent applications include 3dSpAn ( Das *et al.*, 2022) and a spine detector plugin for the Vaa3D software ( Iascone *et al.*, 2020), as well as tools dedicated to two-photon microscopy ( Singh *et al.*, 2017; Rada *et al.*, 2018; Argunşah *et al.*, 2022; Vogel *et al.*, 2023) and structured illumination microscopy ( Kashiwagi *et al.*, 2019). Machine learning based method has also been developed to identify dendritic spines ( Blumer *et al.*, 2015; Smirnov *et al.*, 2018; Guerra *et al.*, 2023), and deep learning approach was used to provide the automated spine segmentation softwares DeepSpineNet and DeepD3 ( Vidaurre-Gallart *et al.*, 2022; Fernholz *et al.*, 2024). Herein, we present an ImageJ plugin that allows spine detection, spine heads segmentation and spine necks tracing. Whilst several tools are available, the general usage of one software for all labeling methods and all image acquisition conditions is unattainable ideal. Hence, our new plugin may be better adapted as compared to others for some users. Spot spine allows precise 3D detection and segmentation of spine heads even in case of high spine density. The automated segmentation ensures reliable and comparable results for shape descriptors of spine head, which are relevant physiological parameters. Furthermore, manual editing at each step of the process ensures that false negative and positive are absent.

## Methods

### Animals

C57BL/6J male mice were maintained in a 12-hour light/12-hour dark cycle, under stable temperature (22°C) and humidity (60%) conditions with ad libitum access to food and water. All experiments were carried out in accordance with the standard ethical guidelines [European Community Council Directive on the Care and Use of Laboratory Animals (86/609/EEC) and the French National Committee (2010/63)]. 

### Neuronal labeling

Fluorescent labeling of dendrites was obtained by diolistic method **(** Heck *et al.*, 2012). DiI (3 mg, ThermoFischer D282) is precipitated on the surface of 1.3 microns tungsten beads (50 mg, BioRad M-20). The coated beads are projected by helium gas pressure (150psi) through a 3 micron pore-size filter (Isopore polycarbonate, Millipore) on brain sections from animals perfused with 1.5% paraformaldehyde. The hydrophobic DiI molecule inserts into plasma membrane and passively diffuse along the dendrite, enabling a fluorescent membrane labeling that outlines neuronal morphology. After labeling, the sections were kept in phosphate buffer saline for 2 hours then mounted in Prolong Gold media (Molecular Probes, P36930).

### Image acquisition and deconvolution

Confocal Laser Scanning Microscope (SP5, Leica) equipped with a 1.4 NA objective (oil immersion, Leica) was used to acquire image stacks with pixel size of 60 nm and z-step of 200 nm, at excitation wavelength of 561 nm and emission range 570-650 nm. Laser intensity was set so that each image occupies the full dynamic range of the detector (low noise Hybrid detector, Leica). Deconvolution with experimental PSF from 175 nm PS-speck Microscope Point Source fluorescent beads using Maximum Likelihood Estimation algorithm was performed with Huygens software (Scientific Volume Imaging). 150 iterations were applied in classical mode, background intensity was averaged from the voxels with lowest intensity, and signal to noise ratio values were set to a value of 20.

## Implementation, features and usage

**Figure 1.**
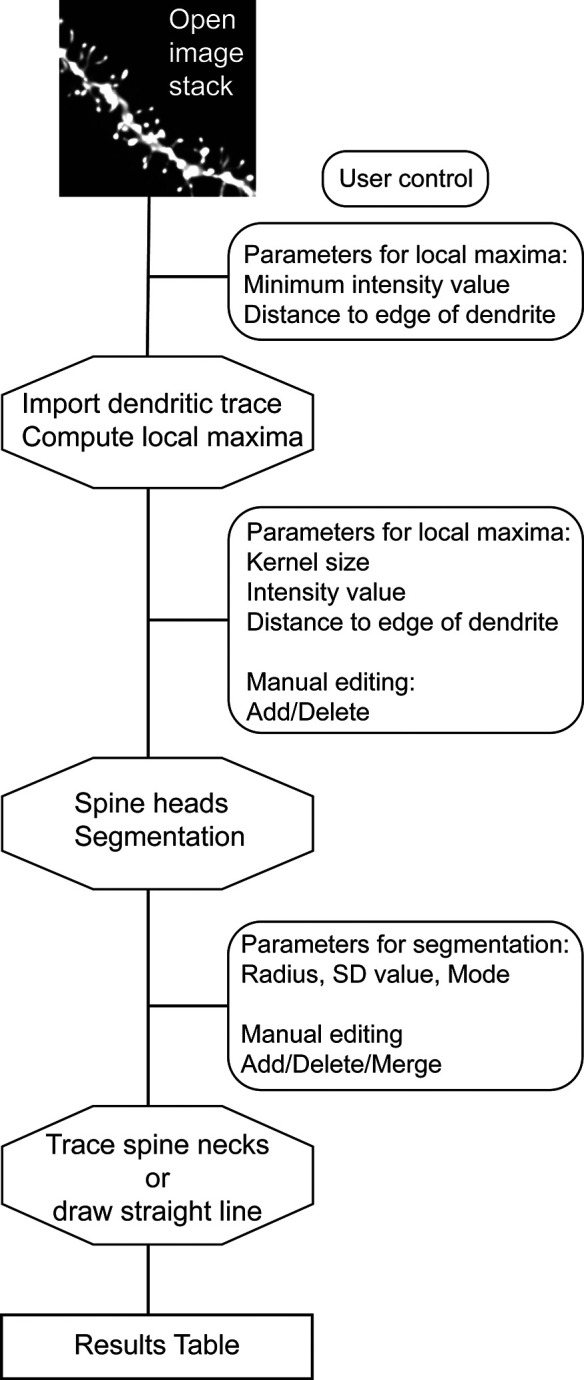
Flowchart of the Spot Spine plugin working process. After an image stack is opened in FIJI/ImageJ and the plugin launched, the user is invited to set parameters for local maxima detection. The plugin imports the dendrite model encoded in a.swc file and compute the local maxima. The user can modify the parameters and manually edit each local maximum. Spine head segmentation is performed by the plugin, and the results can be updated by modifying segmentation parameters as well as manually editing each spine head. The spine necks are then traced, but for images in which the necks are not labeled, the plugin can draw straight lines. A result table is given, including spine head volumes, spine neck length, among other measurements.

Our plugin Spot Spine uses our Spot Segmentation workflow ( Ollion *et al.*, 2013; Heck *et al.*, 2015) and the tracing algorithm from the plugin SNT ( Arshadi *et al.*, 2021) to perform three-dimensional detection and analysis of dendritic spines in image stacks ( Figure 1). The algorithms are adapted to both isotropic and non isotropic voxels, since, typically, microscopy image stacks have lower axial resolution. Spot Spine is implemented as a plugin for ImageJ/FIJI (ImageJ 1.53, Schindelin *et al.*, 2012a, 2012b). The plugin and instructions for use are available at https://imagej.net/plugins/spot-spine.

Before proceeding, it first requires importing a reconstructed model of the dendrite in SWC format. The tracing of the dendrite, coded in a swc file, can be obtained with various freely available tools such as SNT ( Arshadi *et al.*, 2021) or others ( Parekh and Ascoli, 2013; Liu *et al.*, 2022). The dendrite model is automatically imported after launching the plugin. A dendrite coded as SWC is a sequence of connected nodes, thus our plugin applies a frustum between each sphere to improve the representation of the dendrite volume.

**Figure 2.**
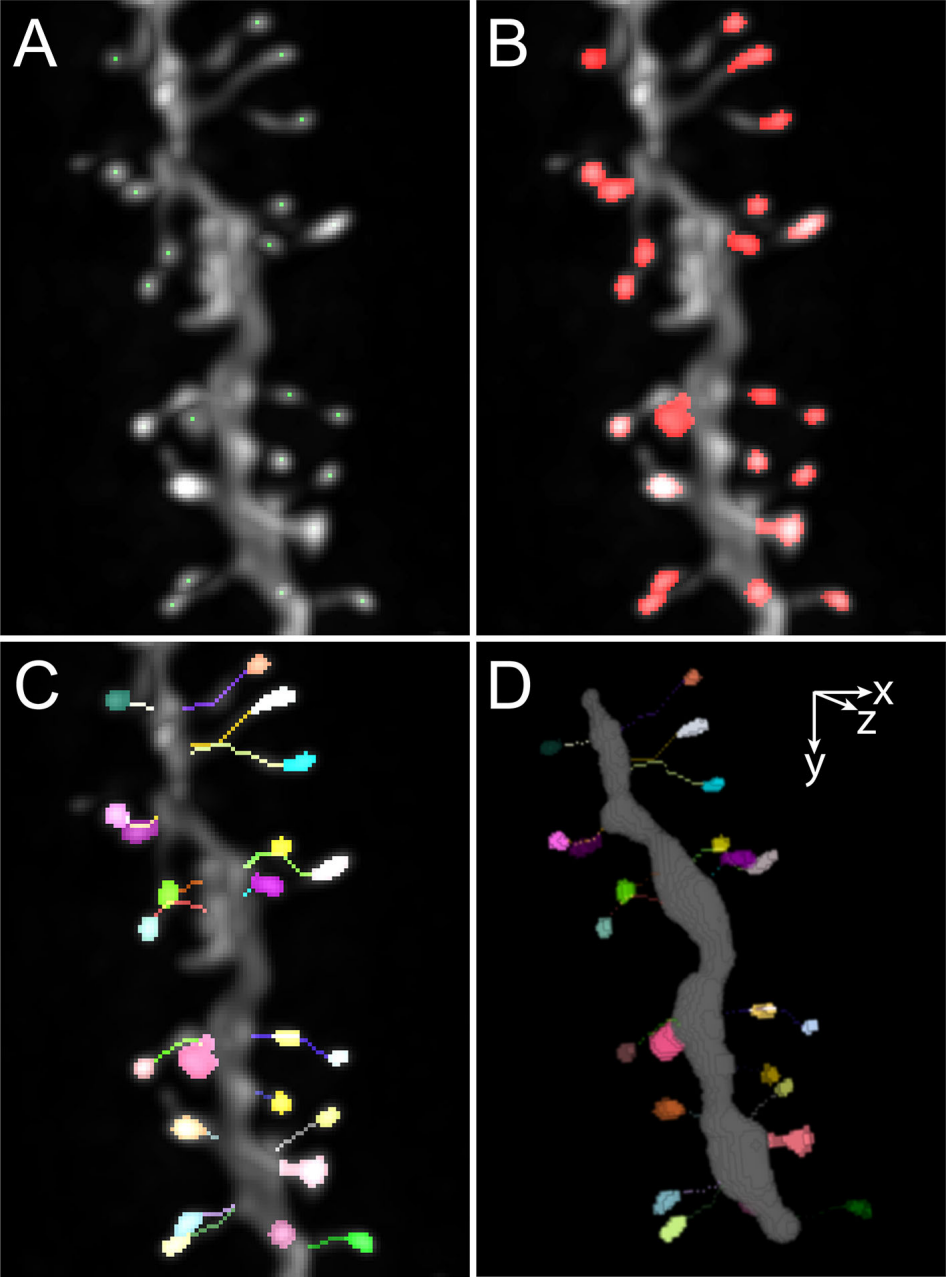
Examples of images illustrating the main steps of the process. A. Maximum intensity projection showing the detected local maxima. B. Maximum intensity projection showing the segmented spine heads. C. Maximum intensity projection showing traced necks and spine heads. D. 3D volume rendering of the dendrite encoded in swc file, segmented spine heads and traced necks.

Spot Spine detects the dendritic spines by computing the local maxima in the neighbouring region of the dendrite. Since some local maxima can be false positives from the background, or spines from another dendrite located near the studied dendrite, the user is invited to define the three following parameters: intensity value underneath which local maxima are ignored, and minimum and maximum distance from the border of the dendrite model, delimiting around the dendrite a 3D region in which local maxima will be computed. The computed local maxima are listed and displayed in the image stack. A maximum projection of the stack is displayed as well, enabling to easily apprehend the content of the image stack ( Figure 2A). The user can then control further intensity and distance criteria to remove false positives. The minimal distance imposed between each local maxima can be reduced or expanded, enabling to adapt to either sparse or dense spine density along the dendrite. Moreover, full manual editing is easily achieved by removing single local maxima or adding local maxima by simply clicking in the image. The manual addition of local maxima is independent of the criteria of intensity and distance. It is noteworthy that the image stack and the maximum projection are synchronized. The user can thus interact on the maximum image projection for obvious cases of false positive and false negative, or in the image stack for better precision. When clicking in the maximum intensity projection, the location of the mouse in the image is recorded, and pixels contained in the region centered around the mouse coordinates are examined in each slice through the depth of the image stack. For spine deletion, the closest maximum is selected based on Nearest neighbor algorithm and deleted from the list. For spine addition, the coordinates of the voxel of highest intensity within a 5x5 region centered around the mouse location is added to the list.

Once the local maxima are found to correspond to each spine head, those are segmented in 3D using the spot segmentation workflow described in Gilles *et al.* (2017). Segmentation is run with set parameters that lead to consistent results with images obtained by confocal and two-photon microscopy from dendrites with membrane and cytosolic labeling, nevertheless, the user can update the segmentation after setting new values for the parameters. The segmented spine heads appear both on the synchronized image stack and maximum intensity projection ( Figure 2B). As described for the selection of local maxima, when clicking in the maximum projection, the object is selected by the analysis of the 3D content of the image stack within a region centered around the 2D coordinates of the mouse location. The user can select spine head that would need to be removed. It is possible to add new spine in case a spine head was not detected by a local maxima by clicking in the spine head. Two spine heads can also be selected to be merged. Indeed, each local maximum will give an object, hence if two local maxima are found in one large spine head, the best strategy is to keep both and thus generate two adjacent objects that can be merged.

In the third step, spine necks are traced using the SNT algorithm ( Figure 2C,D). The minimal euclidian distance between the spine head and the dendrite is computed to identify one voxel at the border of the spine head and one voxel at the border of the dendrite, and the optimal path between these two points is computed using SNT. After tracing, the neck is the one-voxel wide path from the voxel positioned at the edge of the spine head to the voxel preceding the first voxel positioned at the border of the dendrite. Manual editing is provided to delete wrong path and update the neck trace by imposing a new starting point at the border of the spine head. In the case of images in which the necks are not visible, the user can rather choose to obtain the minimal distance between the spine head center and the dendrite which is an estimate of spine length. For each case for which the spine head is in contact with the dendrite, no neck is traced and the spine is categorized as belonging to the stubby type.

After completion of spine detection, spine head segmentation and spine neck tracing, a four-channel image is displayed overlaying the original image, the dendrite coded in the SWC file, the segmented spine heads and the traces of the spine necks. Dendrite legnth, number of spines and spine density is given ina window, and a table is retrieved in which the measurements of several morphological parameters are given, including spine head volume and surface as well as neck length.

## Discussion

We have implemented a new tool for 3D spine detection and analysis as an ImageJ/Fiji plugin. Of note, spine heads detection and segmentation works on 2D images as well, but not the tracing of the necks. To our knowledge, two other ImageJ plugins dedicated to dendritic spines exist: the Dendritic_Spine_Counter for 2D images, and SpineJ which is dedicated to 2D analysis of STED microscopy images ( Levet *et al.*, 2020). Since full automatization is unattainable when considering the wide range of neuronal labeling and image acquisition protocols, manual editing in a user-friendly interface allows to correct spine detection and segmentation. However, most of the procedure remains automated, so spine head segmentation and spine neck tracing retrieve consistent results. One limitation of our plugin is that it does not give any estimate of the spine neck width. We have chosen to avoid the measurement of that parameter because it falls below the resolution of most confocal and two-photon image stacks. Studies specifically dedicated to the spine neck properties may better benefit from custom procedures for spine neck size estimates. An important concern is the signal to noise ratio of the image stacks. Local maxima detection is noise sensitive, hence noise filtering may help to avoid false positive. Deconvolution is generally recommended ( Dumitriu *et al.*, 2011; Heck *et al.*, 2012) since it improves axial resolution, but noise filtering using tools such as the plugin PureDenoise ( Luisier *et al.*, 2010) can also yield good results.

Morphological analysis of dendritic spine is often based on classification into discreet categories, namely stubby, thin, mushroom and filopodia. Stubby spines are devoid of neck, which has functional implication since the neck isolates the spine head from the dendrite. Therefore, the plugin indicates in the result table if the spine is of stubby type. The categorization into thin and mushroom spines has however been shown to be arbitrary, since dendritic spines exhibit a continuum of morphologies ( Wilson *et al.*, 1983; Tonnesen *et al.*, 2014; Ofer *et al.*, 2021, 2022). Filopodia are elongated spine without head which correspond to an immature stage observed during development. Nevertheless, it can be difficult to distinguish filopodia from long thin spines. Therefore, the distribution of unbiased measurement allows an objective assessment of spine morphology ( Pchitskaya and Bezprozvanny, 2020). Categorization of spines by cluster analysis of quantified features have been made ( Luengo-Sanchez *et al.*, 2018; Urban *et al.*, 2019; Kashiwagi *et al.*, 2019). Recent softwares, Dxplorer ( Choi *et al.*, 2023) and SpineTOOL ( Ekaterina *et al.*, 2023), perform a fine analysis of spine morphology by analyzing either 3D surface mesh or chord lens distribution histogram, respectively. The quantitative results obtained with Spot Spine allow the description of the statistical distribution of morphological features, but the user can build categories by grouping dendritic spines according to defined range of values.

## Data Availability

No data are associated with this article.

## Software availability

Software available from: https://imagej.net/plugins/spot-spine

Source code available from: https://github.com/JeffGilles/Spot-Spine/, https://doi.org/10.5281/zenodo.10622975

License: GPL-3.0 - CeCILL
