# Supplementary material for: Spot Spine, a freely available ImageJ plugin for 3D detection and morphological analysis of dendritic spines
Source: F1000Res. 2024 Sep 5;13:176. Originally published 2024 Mar 11. [Version 2] doi: 10.12688/f1000research.146327.2 (PMC11420623; doi:10.12688/f1000research.146327.2)
Supplement: Peer review history

# Reviewer report: Reviewer response for version 2

**DOI:** 10.5256/f1000research.171275.r321507

**Published:** 2024-10-10

**Authors:** Elizabeth McCullagh

**Affiliations:** 1 Oklahoma State University, Stillwater, Oklahoma, USA

This article provides a description of a tool for 3D analysis of dendritic spine morphology linked to a common platform (FIJI) for image analysis in neuroscience. While there are other tools for spine analysis, this tool integrates with the most common image analysis toolbox and is open-source and user friendly while providing importance quantitative measures of spines. I thought that the paper presented the tool very well and included several parameters that were responsive to previous reviewers techniques. The only thing I find lacking is a summary conclusion statement synthesizing the usefulness and application of the tool in neuroscience. Otherwise I think this is an excellent paper and very useful tool.

Are the conclusions about the tool and its performance adequately supported by the findings presented in the article?

Yes

Is the rationale for developing the new software tool clearly explained?

Yes

Is the description of the software tool technically sound?

Yes

Are sufficient details of the code, methods and analysis (if applicable) provided to allow replication of the software development and its use by others?

Yes

Is sufficient information provided to allow interpretation of the expected output datasets and any results generated using the tool?

Yes

Reviewer Expertise:

neurodevelopmental disorders, Fragile X Syndrome, auditory

I confirm that I have read this submission and believe that I have an appropriate level of expertise to confirm that it is of an acceptable scientific standard.

# Reviewer report: Reviewer response for version 2

**DOI:** 10.5256/f1000research.171275.r321505

**Published:** 2024-09-23

**Authors:** Ming Hu

**Affiliations:** 1 Baylor College of Medicine, Houston, Texas, USA

The manuscript and documentation for the software are well written and well organized. The installation process is smooth without any problem. Running Spot Spine with the provided example produced highly similar results as demonstrated in the paper.

 A few minor comments:

 1. Authors claimed in the Results section, the plugin would “allows the analysis of images of dendrites obtained with various labeling and imaging methods”. Given the versatility of ImageJ, this is reasonable. However, the performance would highly be dependent on the SNR. It would be helpful to demonstrate the performance with at least one other labeling/imaging method (such as GFP labeled dendrites).

 2. Comparing the automated segmentation results with one or two of the state-of-art algorithms mentioned in the paper would help to readers know the strength and weakness of the plugin. For example, does this plugin and other algorithm provide comparable auto-segmentation results from the same image? If so, this plugin’s ability to provide manual annotation would be an advantage. Otherwise, the manual annotation part does not have a good basis to start with.

 3. Linking results table with the four-channel overlay image. This part could really help the reader to visually inspect/ assess whether the algorithm/parameters work). I think adding a column (ideally the first column) to indicate the Z index (could be the plane where the CMx, CMy were calculated, or the seed points was) for the detected object would helpful. Also, move the CX, CY, CZ columns to be the first columns.

 4. Expand Instructions (“First step…” in Spot spine’s documentation at its imagej website) on how to prepare new images for processing with the plugin with be helpful.

 Reviewers Zhou & Shan in the first round have raised similar points (1-3), but I am afraid the points should be better addressed.

 Typos:

 “… Dendrite legnth, number of spines and spine density is given ina window..”:

 “legnth” should be “length”,

 “ina” should be “in a”

Are the conclusions about the tool and its performance adequately supported by the findings presented in the article?

Partly

Is the rationale for developing the new software tool clearly explained?

Partly

Is the description of the software tool technically sound?

Yes

Are sufficient details of the code, methods and analysis (if applicable) provided to allow replication of the software development and its use by others?

Yes

Is sufficient information provided to allow interpretation of the expected output datasets and any results generated using the tool?

Yes

Reviewer Expertise:

Systems neuroscience

I confirm that I have read this submission and believe that I have an appropriate level of expertise to confirm that it is of an acceptable scientific standard, however I have significant reservations, as outlined above.

# Reviewer report: Reviewer response for version 2

**DOI:** 10.5256/f1000research.171275.r321504

**Published:** 2024-09-23

**Authors:** Andreas Kist

**Affiliations:** 1 Friedrich-Alexander-University Erlangen-Nürnberg, Bavaria, Germany

The study overall is very nice and I believe that the ImageJ/FIJI plugin will be very useful to others. However, I have some recommendations for the article:

 (i) I've checked the code and would highly appreciate if French comments/documentation would be translated into English, as well as deprecated code (commented variables etc) will be removed for a clean software version.

 (ii) To assess the reliability of the tool, I would recommend to not only show an example, but to also use this dataset to assess the variability/intra- or inter-rater-reliability across individual trials, as settings can be adjusted manually. You could show in a table which measures are affected and how large the estimated error is. This would hint prospective users about the reliability of this method.

Are the conclusions about the tool and its performance adequately supported by the findings presented in the article?

Partly

Is the rationale for developing the new software tool clearly explained?

Yes

Is the description of the software tool technically sound?

Yes

Are sufficient details of the code, methods and analysis (if applicable) provided to allow replication of the software development and its use by others?

Partly

Is sufficient information provided to allow interpretation of the expected output datasets and any results generated using the tool?

Partly

Reviewer Expertise:

Biomedical signal processing, biomedical image analysis and processing, neuroscience

I confirm that I have read this submission and believe that I have an appropriate level of expertise to confirm that it is of an acceptable scientific standard, however I have significant reservations, as outlined above.

# Reviewer report: Reviewer response for version 1

**DOI:** 10.5256/f1000research.160403.r275153

**Published:** 2024-06-07

**Authors:** Georgina M Aldridge

**Affiliations:** 1 Iowa Neuroscience Institute, University of Iowa, Iowa City, USA

The article itself is well written and described, but the software tool is currently difficult. I discuss the article first and the tool second. 

 The article and tool are targeting an important need. Current tools are incredibly expensive and proprietary. Many in the field still use completely manual counting, which precludes unbiased evaluation of volume or shape. The tool description and the article are thus important. 

 For the article, I do recommend the authors say spines are an "estimate" of synaptic number, rather than a "measure". 

 Two other changes are just likely typos, my suggestion in brackets:

 "since the neck represents a morphological constrains [ **constraint**], the spine head is a functional compartment isolated [ **from**] the dendritic shaft"

 "Therefore, quantification of spine density, spine head size and spine neck length are desirable for

 each of these parameters [in order to] retrieve valuable information on neural connectivity, synapse efficacy and plasticity."

 For the tool:

 The FAQ/about text for the tool has multiple typos and should be edited. I was not able to open any of my own 2-photon imaging data sets (despite having an associated SWC file that I made specifically to test this tool). Trying to open them immediately caused an error. The error was not specific so I am unclear what I was doing wrong.

 I was able to open the sample images and tested the program on both a mac and PC running FIJI. The program did find spines in a reliable manner on this one sample image, and produced data that looked exciting and promising. However, as soon as any manual manipulation was attempted, there were difficulties:

 1) Often (on both platforms) the manual edit stopped working. If the wrong button was touched in FIJI it led to two things occurring simultaneously. Restarting and not touching any buttons on FIJI (outside of spot spine) sometimes worked. 

 2) The question mark button did not do anything

 3) Green pixels shown on the max local projection are sometimes not seen on the max local merge file. 

 4) deleting a spine made it so you could not add a spine back at the same location. Often trying to add a spine somewhere else would lead to error "needs a point". 

 5) it was hard to tell when spines are selected- often leading to deleting multiple spines by mistake. There is no undo so this means starting over. Most mistakes I made led to needing to start over.

 6) When getting into the spine neck path, clicking update caused the log to show errors. 

 7) I could not tell which spines were which. I know from the FAQ they are coded by intensity but I could not find how to figure out which was which on the screen. This made it difficult to see how my manual edits were correlating with actual data output. 

 8) Any amount of fussing to try to add or improve a spine manually led to bits of spine all over the dendrite that I couldn't figure out how to edit. 

 Initially- the tool looked quite powerful and did map spines on the one sample image in a way that looked like it might be very useful. Unfortunately, all the manual editing caused disruptions, and this was in a perfect demo image.  If this is due to my own misreading of the instructions, then perhaps a video demonstrating manual editing of spines would help. As with all such tools, it is possible the particulars of my setup made using the tool more difficult, and others might find it more useful. Alternatively, the tool may just need a bit more refinement to be user-ready.

Are the conclusions about the tool and its performance adequately supported by the findings presented in the article?

No

Is the rationale for developing the new software tool clearly explained?

Yes

Is the description of the software tool technically sound?

Yes

Are sufficient details of the code, methods and analysis (if applicable) provided to allow replication of the software development and its use by others?

Yes

Is sufficient information provided to allow interpretation of the expected output datasets and any results generated using the tool?

Yes

Reviewer Expertise:

I have >15 years experience counting dendritic spines. I use FIJI relatively frequently, including to count spines and I am familiar with installing and using plugins, but I am not myself familiar with building/coding plugins.

I confirm that I have read this submission and believe that I have an appropriate level of expertise to confirm that it is of an acceptable scientific standard, however I have significant reservations, as outlined above.

# Response

**Published:** 2024-08-30

**Authors:** Nicolas Heck

**Affiliations:** Neuroscience Paris Seine, Sorbonne Universite, Paris, Île-de-France, France

The article itself is well written and described, but the software tool is currently difficult. I discuss the article first and the tool second. 

 The article and tool are targeting an important need. Current tools are incredibly expensive and proprietary. Many in the field still use completely manual counting, which precludes unbiased evaluation of volume or shape. The tool description and the article are thus important. 

 For the article, I do recommend the authors say spines are an "estimate" of synaptic number, rather than a "measure".

*Response: We agree, modifications have been done in the manuscript.*

 Two other changes are just likely typos, my suggestion in brackets:

 "since the neck represents a morphological constrains [ **constraint**], the spine head is a functional compartment isolated [ **from**] the dendritic shaft"

 "Therefore, quantification of spine density, spine head size and spine neck length are desirable for

 each of these parameters [in order to] retrieve valuable information on neural connectivity, synapse efficacy and plasticity."

*Response: Thank you, the manuscript has been corrected.*

 For the tool:

 The FAQ/about text for the tool has multiple typos and should be edited.

*Response: Thank you for pointing that out, we will correct the webpage*

 I was not able to open any of my own 2-photon imaging data sets (despite having an associated SWC file that I made specifically to test this tool). Trying to open them immediately caused an error. The error was not specific so I am unclear what I was doing wrong.

*Response: Opening images files is completely independent of our plugin. Opening images is integral part of FIJI/ImageJ. For proprietary files such as .lif or .czi coming from commercial microscopes, the plugin LOCI Importer is needed, however the plugin is by default included in FIJI. In any case, our plugin starts after an image has been opened so the problem is unrelated to our plugin.*

*Or you mean that after the image was open, you could not run spot spine. It could come from the swc file. Some swc contain the xyz coordinates and the diameter of spheres along the dendrite. Some swc contain only the xyz coordinates thus coding a line. When you tested our plugin, it was not able to work with swc without diameter estimation of the dendr* *ite. We have updated the plugin so both type of swc file are compatible.*

 I was able to open the sample images and tested the program on both a mac and PC running FIJI. The program did find spines in a reliable manner on this one sample image, and produced data that looked exciting and promising. However, as soon as any manual manipulation was attempted, there were difficulties:

 1) Often (on both platforms) the manual edit stopped working. If the wrong button was touched in FIJI it led to two things occurring simultaneously. Restarting and not touching any buttons on FIJI (outside of spot spine) sometimes worked.

*Response: The other reviewer and some colleagues did not report similar problems. It may be related to the ImageJ or FIJI version used, or to the version of the plugins 3DImageSuite or SNT installed. A more detailed description of the problem is required to fully respond about the problem mentionned here. For our previously published plugin DiAna, as well as for Spot Spine, our email adress is on the webpage of the plugin and in the publication. We keep answering to all request and help users. In addition, we answer to questions posted on the widely used FIJI/ImageJ oriented « scientific community image forum » : https://forum.image.sc.*

 2) The question mark button did not do anything

*Response: Thank you for pointing that out. We updated the plugin so the question mark drives the user to the spot spine webpage. The question mark at the segmentation step drives the user to the webpage describing the segmentation method.*

 3) Green pixels shown on the max local projection are sometimes not seen on the max local merge file.

*Response: Unfortunately, green pixels can be difficult to see depending on the local intensity in the other channel. Any plugin can only use the look-up tables and image display internally coded in FIJI/ImageJ. In the plugin, it is offered to have the image in either blue or gray levels. Nevertheless, the user can go to Image>Look-up tables and apply any of those while running the plugin. In some cases, changing brightness&contrast will help as well (Image>Adjust>Brightness&Contrast)*

 4) deleting a spine made it so you could not add a spine back at the same location. Often trying to add a spine somewhere else would lead to error "needs a point". 

*Response:In an earlier version of the plugin, it was not possible to add a spine in a head that was previously deleted. We have updated the plugin so it is know possible.*

*The message « needs a point » indicates that it is first required to define a region of interest before clicking on the « Add » button. As written in the dialog window, the user holds the SHIFT key and click in the image. Then a region of interest appears (as a square selection), and then clicking on « Add » button will prompt the plugin to find a local maxima within the region, and segment the object.*

 5) it was hard to tell when spines are selected- often leading to deleting multiple spines by mistake. There is no undo so this means starting over. Most mistakes I made led to needing to start over.

*Response: Undo function is not build in FIJI/ImageJ, it is a known limitations of the program which is not in our hands. Also build in FIJI/ImageJ, selection is shown as yellow line around the selected objet. It is true that once selecting a spine head, to select another will not unselect the previously selected one. The simple reason is that is allows to merge objects when two objects are found in one single spine head (a needed function since in some large spine heads, more than one local maximum can be found, hence the merge function allows to overcome oversegmentation). Once the merge is performed, no spine head is selected.*

*To unsure no objects are selected, the user can click outside of a spine head. This will by default unselect all selected spines. It is a build in function of FIJI/ImageJ, that when clicking outside a selection or an object, a « select none » function is applied.*

 6) When getting into the spine neck path, clicking update caused the log to show errors.

*Response: It is difficult to respond without knowing the content of the error message. For our previously published plugin DiAna, as well as for Spot Spine, our email adress is on the webpage of the plugin and in the publication. We keep answering to all request and help users. In addition, we answer to questions posted on the widely used FIJI/ImageJ oriented « scientific community image forum » : https://forum.image.sc.*

*Concerning updating the necks. First delete a neck by clicking on (or near) the head or the neck and then click on « delete path ». Then click again in head while holding SHIFT key, a yellow dot appears. Then click on « Update path ». A new path starting from the dot will be created. Please note that the algorithm computes best path it can detect, so the new path may be same as first. Neck detection is very challenging because intensity of the neck in the image is usually very low. Anytime delete or update path function is applied, the results Table is updated.*

 7) I could not tell which spines were which. I know from the FAQ they are coded by intensity but I could not find how to figure out which was which on the screen. This made it difficult to see how my manual edits were correlating with actual data output.

*Response: We acknowledge this would help, but annotation on the output image is not straightforward. If in the image stack, it would be visible only in one plane ; if on the maximum projection numbers would overlap if spines are close to each other.*

*Nonetheless, the user has several options because the ID of the spine in the table is color coded in the image. For a spine with ID 25, the voxels in the image will have an intensity of 250. Therefore :*

*The user can point on the spine in the image and read the intensity (= the ID) in the FIJI/ImageJ menu window. Since the image is a composite with four channels, the user needs to select the spine head channel (i.e. first channel) otherwise the intensity given may be 0 (in necks and dendrite channels) or a number which is the gray level of the orignial image (fourth channel). To select a channel see the « C » sliding bar at the bottom of the image.*

*The user can read an ID in the table and find it by checking the intensities of the spines in the image. The ID numbers increase with stack depth.*

*Another way is to use the 3DManager of the plugin 3DImageSuite (that plugin is necessarily installed for the proper functioning of Spot spine). In short, the steps to follow are : Go to plugin menu, 3DSuite, 3DManager. Once the manager is open, click on the composite image containing the spine heads, necks, dendrite and image to activate. Choose the spine head channel (i.e. first channel). In the 3DManager, click on « Add Image ». All objects will be listed in the manager. Names are obj1-val10 ; obj2-val20 ; obj3-val30 and so on. ObjX is same as in « Name » column of the Result Table. ValX is the same as the spine head intensity in the image. Click on « Live ROI ». Click on one ROI in the list to make it encircled in yellow in the image stack.*

 8) Any amount of fussing to try to add or improve a spine manually led to bits of spine all over the dendrite that I couldn't figure out how to edit. 

*Response: It is difficult to respond without seeing an image on which the phenomenon you described appears. Again, for our previously published plugin DiAna, as well as for Spot Spine, our email adress is on the webpage of the plugin and in the publication. We keep answering to all request and help users. In addition, we answer to questions posted on the widely used FIJI/ImageJ oriented « scientific community image forum » : https://forum.image.sc.*

# Reviewer report: Reviewer response for version 1

**DOI:** 10.5256/f1000research.160403.r275149

**Published:** 2024-05-22

**Authors:** Jiang-Ning Zhou, Qing-Hong Shan

**Affiliations:** 1 Institute of Brain Science, The First Affiliated Hospital of Anhui Medical University, Hefei, Anhui, China

The authors of this article present a plugin specifically designed for three-dimensional spine analysis on the ImageJ platform. They provide detailed explanations of the underlying principles and usage procedures of the plugin, and compare it with other software for 2D or 3D dendritic spine analysis, such as Dendritic_Spine_Counter, SpineJ, Dxplorer, and SpineTOOL. The article is user-friendly and provides comprehensive details to comprehend the described methods and their practical application. Following the authors' meticulous instructions, we effortlessly installed and utilized the plugin. The manuscript fulfills expectations for a software paper; however, while reading the paper and using the software, some minor suggestions and questions have emerged.

 1. In the "Introduction" section, the authors present various software and methods for spine analysis; however, they fail to provide a clear rationale behind the development of the Spot Spine Plugin, despite the existence of numerous available techniques. Please provide a brief introduction here regarding the purpose and significance of developing this plugin.

 2. On the interface for selecting spine detection parameters, in order to enhance users' comprehension of parameter definitions, could you please specify the units for "Minimum distance to dendrite" and "Maximum distance to dendrite" (in pixels or microns)?

 3. The "Result Table" generated by the plugin presents a wide range of parameters; however, it has come to my attention that the commonly utilized parameter of dendritic spine density is conspicuously absent. It is strongly recommended to incorporate statistical data for this particular parameter.

 4. The statistical parameters include the identification (ID) of each identified spine; however, the corresponding ID is not annotated on the output images, which poses challenges for traceability. It is recommended to incorporate ID markers in the output images to facilitate referencing.

 5. The statistical analysis of dendritic spines often necessitates the processing of a large number of dendrites, and batch analysis proves to be highly efficient in this regard. It has been observed that Spot Spine lacks a batch analysis feature, thus it is recommended to incorporate this functionality into the plugin.

 6. The article utilizes the analysis of spines on a single dendrite as an illustrative example. It would be beneficial to provide clarification and discussion regarding the applicability of the plugin to dendrites with multiple branches.

Are the conclusions about the tool and its performance adequately supported by the findings presented in the article?

Yes

Is the rationale for developing the new software tool clearly explained?

Partly

Is the description of the software tool technically sound?

Yes

Are sufficient details of the code, methods and analysis (if applicable) provided to allow replication of the software development and its use by others?

Yes

Is sufficient information provided to allow interpretation of the expected output datasets and any results generated using the tool?

Yes

Reviewer Expertise:

neuroscience

We confirm that we have read this submission and believe that we have an appropriate level of expertise to confirm that it is of an acceptable scientific standard.

# Response

**Published:** 2024-08-30

**Authors:** Nicolas Heck

**Affiliations:** Neuroscience Paris Seine, Sorbonne Universite, Paris, Île-de-France, France

The authors of this article present a plugin specifically designed for three-dimensional spine analysis on the ImageJ platform. They provide detailed explanations of the underlying principles and usage procedures of the plugin, and compare it with other software for 2D or 3D dendritic spine analysis, such as Dendritic_Spine_Counter, SpineJ, Dxplorer, and SpineTOOL. The article is user-friendly and provides comprehensive details to comprehend the described methods and their practical application. Following the authors' meticulous instructions, we effortlessly installed and utilized the plugin. The manuscript fulfills expectations for a software paper; however, while reading the paper and using the software, some minor suggestions and questions have emerged.

 1. In the "Introduction" section, the authors present various software and methods for spine analysis; however, they fail to provide a clear rationale behind the development of the Spot Spine Plugin, despite the existence of numerous available techniques. Please provide a brief introduction here regarding the purpose and significance of developing this plugin.

*Response: We have updated the introduction to better explain the rationale for proposing Spot Spine.*

*« Whilst several tools are available, the general usage of one software for all labeling methods and all image acquisition conditions is unattainable ideal. Hence, our new plugin may be better adapted as compared to others for some users. Spot spine allows precise 3D detection and segmentation of spine heads even in case of high spine density. The automated segmentation ensures reliable and comparable results for shape descriptors of spine head, which are relevant physiological parameters. Furthermore, manual editing at each step of the process ensures that false negative and positive are absent. »*

 2. On the interface for selecting spine detection parameters, in order to enhance users' comprehension of parameter definitions, could you please specify the units for "Minimum distance to dendrite" and "Maximum distance to dendrite" (in pixels or microns)?

*Response:Thank you for that suggestion. We updated the plugin so that information appears.*

 3. The "Result Table" generated by the plugin presents a wide range of parameters; however, it has come to my attention that the commonly utilized parameter of dendritic spine density is conspicuously absent. It is strongly recommended to incorporate statistical data for this particular parameter.

*Response:The Result Table gives information on each spine, but spine density is obviously essential information. It actually was in the plugin, but your commentary made us realize that the code was missing in the version uploaded on the webpage. The plugin has now been updated so at the end of the process (after clicking « finish »), the following informations appears in the Log window : image file name, dendrite length ; total number of spines ; number of spines with neck ; number of stubby spines ; total spine density for 10micron of dendritic length ; spine with neck density for 10micron of dendritic length ; stubby spine density for 10micron of dendritic length. Since it was not mentionned in the manuscript, we added the following sentence is the results section : « Dendrite length, number of spines and spine density is given in a log window ».*

 4. The statistical parameters include the identification (ID) of each identified spine; however, the corresponding ID is not annotated on the output images, which poses challenges for traceability. It is recommended to incorporate ID markers in the output images to facilitate referencing.

*Response: We acknowledge this would help, but annotation on the output image is not straightforward. If in the image stack, it would be visible only in one plane ; if on the maximum projection numbers would overlap if spines are close to each other.*

*Nonetheless, the user has several options because the ID of the spine in the table is color coded in the image. For a spine with ID 25, the voxels in the image will have an intensity of 250. Therefore :*

*The user can point on the spine in the image and read the intensity (= the ID) in the FIJI/ImageJ menu window. Since the image is a composite with four channels, the user needs to select the spine head channel (i.e. first channel) otherwise the intensity given may be 0 (in necks and dendrite channels) or a number which is the gray level of the orignial image (fourth channel). To select a channel see the « C » sliding bar at the bottom of the image.*

*The user can read an ID in the table and find it by checking the intensities of the spines in the image. The ID numbers increase with stack depth.*

*Another way is to use the 3DManager of the plugin 3DImageSuite (that plugin is necessarily installed for the proper functioning of Spot spine). In short, the steps to follow are : Go to plugin menu, 3DSuite, 3DManager. Once the manager is open, click on the composite image containing the spine heads, necks, dendrite and image to activate. Choose the spine head channel (i.e. first channel). In the 3DManager, click on « Add Image ». All objects will be listed in the manager. Names are obj1-val10 ; obj2-val20 ; obj3-val30 and so on. ObjX is same as in « Name » column of the Result Table. ValX is the same as the spine head intensity in the image. Click on « Live ROI ». Click on one ROI in the list to make it encircled in yellow in the image stack.*

 5. The statistical analysis of dendritic spines often necessitates the processing of a large number of dendrites, and batch analysis proves to be highly efficient in this regard. It has been observed that Spot Spine lacks a batch analysis feature, thus it is recommended to incorporate this functionality into the plugin.

*Response: In nearly all cases, a fully automated procedure would bring false positives and false negatives. Hence we made the choice to design a tool that allows manual editing at each step of the process. Therefore a batch mode would not be of much help. Nevertheless, if several users would contact us for expressing that need, it could be implemented.*

 6. The article utilizes the analysis of spines on a single dendrite as an illustrative example. It would be beneficial to provide clarification and discussion regarding the applicability of the plugin to dendrites with multiple branches.

*Response: If the swc file encode a dendrite splitting in two branches (or more), the plugin will detect spines all along the dendrite. In principle, the plugin could be used to detect spines on a complete dendritic tree if the image has the sufficient resolution. However, the purpose of the plugin not to analyse dendritic tree morphology, hence the spine density will not be given for each branch, but as one value for a total dendrite length.*
